# The importance of examining the Hardy-Weinberg Equilibrium in genetic association studies

**DOI:** 10.22099/mbrc.2023.48386.1872

**Published:** 2024

**Authors:** Mostafa Saadat

**Affiliations:** Department of Biology, School of Science, Shiraz University, Shiraz, Iran

The objective of a genetic association study, is to determine whether a potential genetic polymorphism contributes to an individual's susceptibility to a particular disease or characteristic. In scientific way, an association is typically described as a statistically significant difference between two groups (case and control) concerning a set of study variables. In genetic association studies, researchers examine the genotypic and allelic frequencies of a particular polymorphism in both the case and control groups to identify any possible associations. STrengthening the REporting of Genetic Association Studies (STREGA) [1]. recommends that researchers investigate the Hardy-Weinberg Equilibrium (HWE) through the examination of their control subjects' genotypes. According to HWE, there should not be any significant difference between the observed and expected values for genotypes of a given genetic polymorphism in a large population with random mating, and in the absence of mutation, migration, and natural selection. Deviation from these assumptions resulted in observed and expected value differences that may have reached statistical significance. 

The presence of evolutionary factors, such as mutation, migration, natural selection, genetic drift, and non-random mating, can result in a noteworthy difference between the anticipated and observed genotypic outcomes. It should be noted that this is not the only factor at play and other factors contribute as well. Studies indicate that researchers’ mistakes, like genotyping errors, are commonly responsible in such instances. Another critical error is the inclusion of individuals from at least two separate gene pools in the study groups, which results in a sampling error. This undermines trust in the comparison of genotypic frequencies between the case and control groups. 

The main purpose of comparing observed and expected genotypic frequencies under the Hardy-Weinberg equilibrium is to assist researchers in recognizing potential issues with their work. Researchers can subsequently take corrective action to address identified errors (genotyping / sampling errors). Regrettably, some researchers fail to compare observed and expected frequencies, make calculation errors, or fail to consider statistical significance. Many of them are unaware that such oversimplification leads to a significant number of published articles that lack credibility. Accordingly, it is of utmost importance that studies presenting findings adhere to strict standards. Since the issuance of the STREGA statement in 2009, there has been no decline in the occurrence of this issue in genetic association study reports. It is essential for researchers interested in genetic association studies to compare the observed and HWE-expected genotypic values, as stated in the STREGA statement. It is hoped that following the recommendations of the STREGA statement will improve the quality of genetic association studies.

## Declaration of competing interest:

None

## References

[1] Little J, Higgins JP, Ioannidis JP, Moher D, Gagnon F, von Elm E, Khoury MJ, Cohen B, Davey-Smith G, Grimshaw J, Scheet P, Gwinn M, Williamson RE, Zou GY, Hutchings K, Johnson CY, Tait V, Wiens M, Golding J, van Duijn C, McLaughlin J, Paterson A, Wells G, Fortier I, Freedman M, Zecevic M, King R, Infante-Rivard C, Stewart A, Birkett N (2009). STrengthening the REporting of Genetic Association studies (STREGA)--an extension of the STROBE statement. Eur J Clin Invest.

